# Taste perception in children with different caries activity

**DOI:** 10.1007/s40368-022-00739-1

**Published:** 2022-08-09

**Authors:** S. Hertel, L. Mühlig, C. Hannig, T. Hummel

**Affiliations:** 1 Clinic of Operative Dentistry, Medical Faculty Carl Gustav Carus, Technische Universität Dresden, Fetscherstraße 74, 01307 Dresden, Germany; 2 Interdisciplinary Center Smell and Taste, Department of Otorhinolaryngology, Medical Faculty Carl Gustav Carus, TU Dresden, Fetscherstrasse 74, 01307 Dresden, Germany

**Keywords:** Taste perception, Caries, Children, Dietary habits

## Abstract

**Purpose:**

The aim of the study was to investigate whether caries-active and caries-free children differ in terms of their taste perception for sweet, sour, salty and bitter.

**Methods:**

The study group consisted of 100 children aged 5–10 years: 50 caries-free children and 50 children with at least four untreated carious lesions. Taste perception was analysed using two test systems. First, filter paper strips impregnated with four taste qualities (sweet, sour, salty, and bitter) at four defined intensities were used (“taste strips”). Then a tasting spray in suprathreshold concentration of the respective taste was applied. The children were asked to name the perceived taste. The parents answered a questionnaire about the children’s dietary habits.

**Results:**

The children with high caries activity scored lower on average on the taste strips than the caries-free participants. For the taste sprays, the perception of the taste quality “bitter” was significantly worse in the children with caries than in caries-free children (Mann–Whitney *U* test *p* < 0.05).

**Conclusion:**

The results of this study suggest that taste preferences in children are associated with increased susceptibility to tooth decay.

## Introduction

The high prevalence of tooth decay in children worldwide continues to be a serious health problem (Kassebaum et al. 2015). Dental caries is caused by the metabolization of carbohydrates by cariogenic microorganisms on the tooth surface (Fejerskov 2004). Various environmental factors such as dental hygiene, regular dental check-ups, socio-economic status, general health and cultural background are involved in the extent of caries in children (Corrêa-Faria et al. 2016). In fact, the distribution of caries in children is skewed; while a large proportion of children have no caries, especially children from socially weaker families have a high caries burden (Koch et al. 2017; Petersen, 2003). Sucrose consumption is one of the most important aetiological factors in the development of caries. On the one hand, cariogenic microorganisms such as *Streptococcus mutans* (*S. mutans*) can metabolize sucrose for energy. On the other hand, bacterial glucosyltransferases produce glucans that enable the formation of a three-dimensional plaque matrix on the tooth surface (Bowen and Koo 2011; Grychtol et al. 2015).

For children, taste is among the important driving forces for food consumption. A large part of children’s daily energy comes from sugary foods and soft drinks (Abhiram et al. 2019). It is assumed that the perception of the sweet taste has an influence on the intake of sucrose and thus on the development of caries (Alanzi et al. 2013; Jurczak et al. 2020; Sundin and Granath 1992). Several studies have been conducted focusing on sweetness preference, frequency of sugar intake and the levels of caries (Ashi et al. 2017; Bretz et al. 2006). A multicenter cross-sectional study by Ashi et al*.* investigated the sweet taste perception and dental caries in 669 schoolchildren (220 Italian, 224 Mexican, and 225 Saudi Arabian children). A positive correlation was found between sweet taste perception and manifestation of caries in all three countries (Ashi et al. 2017).

In a recent study by Jurczak et al. the taste perception of sweet was compared between children with caries and without caries. The sweet taste perception of children with caries was characterized by a lower susceptibility to sucrose (Jurczak et al. 2020). Further, a positive correlation between the perception of sweet taste and the occurrence and intensity of the cariogenic process was found (Jurczak et al. 2020).

A connection between genetically determined taste sensitivity and caries has also been described (Alkuhl et al. 2022). A preference for bitter and sweet tastes is based on taste sensitivity to certain bitter substances and was found to be associated with variation in food preferences in children (Alanzi et al. 2013; Alkuhl et al. 2022).

The studies to date concerning the relationships between taste perception and caries focus on the taste qualities sweet and bitter. Much less is known about the taste perception of sour or salty in children with different caries status. The aim of this study was to investigate differences in taste perception of sweet, sour, salty and bitter in children with caries compared to caries-free children. The hypothesis was that children with caries are, overall, less sensitive to taste qualities than dentally healthy children.

## Materials and methods

A total of 100 children aged 5–10 years participated in the study. This number was assumed to provide meaningful results based on a sample size estimation for two independent groups considering a relatively large effect size (alpha 0.05, two-sided testing, effect size 0.8, power 0.95—sample size estimate of 42 per group) (Faul et al. 2009).

Fifty subjects were caries-free with no decayed, missing or filled teeth (dmf/*t* = 0) and 50 children had at least 4 untreated carious lesions (dmf/*t* > 4). The number of subjects was based on previous studies (Hummel et al. 2009; Schriever et al. 2021). The caries-active children were recruited at the Clinic of Operative and Paediatric Dentistry, Medical Faculty Carl Gustav Carus in Dresden. They were referred to the clinic by their dentist for dental treatment under general anaesthesia due to multiple carious lesions. The children usually came from socially weaker families with a low educational status. The caries-free children were recruited in a primary school in Bad Schlema (Saxony). A dentist experienced in paediatric dentistry performed a dental examination and dmf/t values were calculated. All subjects were of good general health. Both parents and children gave written informed consent to participate in the study. The Ethics Committee of the Medical Faculty at the TU Dresden (EK # 532112020) approved the study design.

Two tests were applied to investigate the children’s taste perception. All children started with the well validated taste strips which are filter paper strips impregnated with one of the four tastants sweet, sour, salty and bitter at four different concentrations each (Table 1) (Landis et al. 2009; Mueller et al. 2003).

**Table 1:** Concentrations of the taste substances in the test strips and test sprays

Taste	Sweet	Sour	Bitter	Salty
Substance	Sucrose	Citric acid	Quinine hydrochloride	Sodium chloride
Concentrations taste strips [g/ml]	0.05 0.1 0.2 0.4	0.05 0.09 0.165 0.3	0.0004 0.0009 0.0024 0.006	0.016 0.04 0.1 0.25
Concentrations spray [g/10 g water]	1	0.5	0.005	0.75

The children were each asked to place a test strip on the centre of their tongue, close their mouth and suck on the strip. Then the perceived taste should be named. To this end, a flash card with the symbols of the four possible tastes (lemon—sour, candy—sweet, salt cracker—salty, and cough syrup—bitter) was presented to the children to help them identify the tastant. The taste strips were applied in a predetermined randomised order of increasing intensities. There was a short break between the test strips, the children did not rinse with water in between.

A second suprathreshold taste test was conducted using “taste sprays” of the four respective tastes (Table 1) (Welge-Lüssen et al. 2011). Each spray was only applied once. The participants opened their mouth, put out their tongue and a spray with a volume of approximately 150 μl was applied. Then the children took their tongue inside their mouth and moved it. The taste had to be identified as either sweet, sour, salty or bitter (Welge-Lüssen et al. 2011).

The entire procedure to test the four tastes required approximately 10 min. During the test, parents were asked to complete a questionnaire about their children’s dietary habits.

### Statistical analysis

The statistical analysis was performed with SPSS (Statistical Packages for Social Sciences, version 27.0, SPSS Inc., Chicago, IL, USA). Comparison between the two groups was calculated using *t* test, Mann–Whitney *U* test and Chi-square test. Correlation analyses were performed according to Pearson. *p* values lower than 0.05 were considered significant.

## Results

The baseline data for the caries-active and caries-free children are presented in Table 2. There were no significant differences in the average age or gender distribution of the two groups.

**Table 2:** Number, gender distribution, average age and caries index of the two study groups

	Caries-free group	Caries group
*n*	50	50
Gender	24 ♂; 26 ♀	29 ♂; 21 ♀
Mean age	7.7 ± 1.2	7.4 ± 1.3
Mean dmf/*t*	0	6.8 ± 2.7
Mean dmf/s	0	15.4 ± 10.3

**Fig. 1:**
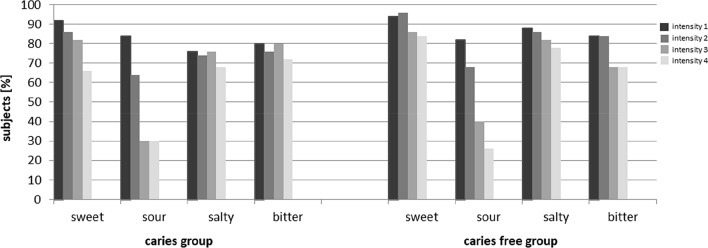
Results of the test strips for caries- and caries-free children: number of participants in percent who correctly named the tastes in descending intensity

For the taste strip test, no significant differences were detected in taste perception between children with caries and caries-free subjects (Fig. 1). However, on average children with caries scored lower than the caries-free participants. In addition, more children from the caries group tended to score 8 and less on the taste strip test compared to the caries-free children (caries-group: 18% vs. caries-free group: 8%; Chi test: *p* = 0.23).

**Fig. 2:**
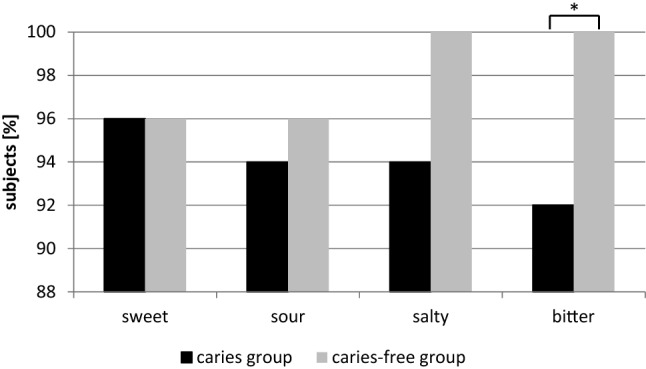
Results of the test sprays for caries- and caries-free group: number of participants in percent who correctly identified the test sprays. For bitter, statistically significant differences were found (Mann–Whitney *U* test *p* < 0.05). Please note that the Y-axis starts at 88%

In the test sprays, the taste quality bitter was named correctly more often by the caries-free children (Fig. 2). This difference was statistically significant (*p* < 0.05). A significant correlation was found between dmf/t/dmf/s values and spray test (Pearson correlation; *r* = − 0.39 *p* < 0.01). The evaluation of the questionnaire on the children's drinking habits and consumption of sweets only showed a significant difference between the study groups in the consumption of juice spritzer (Chi test: *p* = 0.04) (Tables 3 and 4).

**Table 3:** Evaluation of the questionnaire on the drinking habits of the participating children

Drinking habits	Caries group (*n* = 50)				Caries-free group (*n* = 50)				*p* value
	Very often [%]	Often [%]	Rarely [%]	Never [%]	Very often [%]	Often [%]	Rarely [%]	Never [%]	
Water	54	24	18	4	64	26	10	0	0.30
Juice	10	10	60	20	2	14	68	16	0.32
Juice spritzer	18	30	40	12	8	14	54	24	0.04
Sweetened tea	4	14	36	46	4	2	38	56	0.16
Unsweetened tea	14	32	28	26	18	34	32	16	0.65
Cocoa	8	26	46	20	6	30	58	6	0.19
Iced tea	2	0	28	70	0	2	24	74	0.53
Lemonade/Cola	2	12	48	38	0	6	48	46	0.49

Statistical evaluation was performed with Chi-square test

**Table 4:** Evaluation of the questionnaire on the consumption sweets of the participating children

Sweet Consumption	Caries group (*n* = 50)					Caries-free group (*n* = 50)					*p* value
	Several times a day [%]	Daily [%]	Once a week [%]	Several times a week [%]	Never [%]	Several times a day [%]	Daily (%]	Once a week [%]	Several times a week [%]	Never [%]	
Gummi Bears	2	14	40	34	8	2	4	46	42	6	0.47
Candies	0	8	8	40	44	0	6	24	36	32	0.16
Chocolate	0	8	22	36	18	0	4	50	42	4	0.10
Sweet snacks				42	28	0	6	28	22	42	0.22
Ice cream	2	0	16	66	16	0	4	32	48	16	0.45
Salt biscuits	2	0	16	66	16	0	0	52	56	30	0.29

Statistical evaluation was performed with Chi-square test

## Discussion

The current study investigated the taste perception of the taste qualities sweet, sour, salty and bitter in children with caries and caries-free children. It was assumed that children with caries are less sensitive for taste perception. Our results support the assumption for the taste quality of bitter as we found out with the spray test.

The use of taste strips is an established method in the investigation of taste perception (Landis et al. 2009; Manzi and Hummel 2014). The strips allow the presentation of stimuli in the form of a dried taste at different concentrations. Children were asked to identify sweet, sour, salty and bitter taste strips; association of tastes with flash cards has also have been used successfully in previous studies (Heckmann et al. 2005; Manzi and Hummel 2014). These concentrations were chosen so that the highest concentration is recognised by almost all healthy participants. In the present study, although the caries-free group had higher average scores than the caries-active group, there was no statistically significant difference in the accuracy of identifying the taste qualities. Similar to previous studies, the children of both groups commonly mistook bitter and sour for salty and salty for sour (Manzi and Hummel 2014; Welge-Lüssen et al. 2011). Sweet was identified with the highest success rate. The low accuracy in the perception of sour taste in this study is in line with previous work (Manzi and Hummel 2014) and could be seen regardless of caries activity in both study groups.

Our study found a reduced perception of the suprathreshold taste quality bitter in children with caries. A previous study on taste perception in children by Furquim et al. also observed a reduced ability to taste bitter, while no significant difference was found in the perception of sweet between the low and high caries severity groups (Furquim et al. 2010). There is a genetic variation in taste receptors that is associated with differences in food choices and ultimately with caries susceptibility. One such genetic factor is the perception of the bitter taste, which is mediated by the gene TAS2R38. The sensitivity to bitter substances such as 6-n-propylthiouracil (PROP) is mediated by this gene (Lumeng et al. 2008; Pidamale et al. 2012). PROP is a pharmacological drug originally used in the treatment of Grave’s disease, which is used to determine the sensitivity levels to bitter and sweet taste (Alkuhl et al. 2022). Subjects are largely categorized according to their ability to taste the bitterness of PROP, to so-called “super-tasters” who perceive PROP as extremely bitter, medium tasters who experience bitterness at lower intensity, and non-tasters who do not perceive the bitter taste of PROP (Alanzi et al. 2013; Alkuhl et al. 2022; Garcia-Bailo et al. 2009). It was shown that the majority of PROP non-tasters like sweet foods and prefer strong tasting foods, while the majority of PROP supertasters tend to dislike sweet foods (Alanzi et al. 2013; Hedge and Sharma 2008). Several studies show that caries experience in children was significantly higher for non-tasters than supertasters (Alanzi et al. 2013; Lin, 2003; Rupesh and Nayak 2006). Further, the relationship between mothers’ taste preference (PROP-taster/non-taster) and the caries experience of their children was shown (Abhiram et al. 2019; Alanzi et al. 2013). Hence based on their lower performance in identification of the bitter taste spray it can be assumed that the caries-active children in our study belong to the non-taster group.

One limitation of our study may be the wide age range of our study groups. Five-year-old children may still have too low cognitive abilities to correctly assign the taste qualities. The socio-economic background could also influence the results. While the children with caries were predominantly from socially weak families with a low level of education, the caries-free children were from families from the entire population spectrum of a provincial town in Germany. When the parents answered the questionnaires on the dietary habits of the study participants, there was no statistically significant difference between the two groups except for the consumption of juice spritzer, whereby the caries-active children tended to eat sweeter food. It can be assumed that the parents of the caries-free children answered honestly, while the parents of the caries-active children may have glossed over the information. With regard to the reduced perception of the taste quality bitter in the caries-active children, it can be assumed that caries-free children have already consciously consumed bitter tastes in healthy foods such as salads, Brussels sprouts or broccoli through a balanced diet. Food preferences are formed in childhood and often persist beyond that age. Foods rich in energy, sugar and salt are preferred. Although there are genetically determined individual differences, the repeated offering of certain foods and the eating habits in the immediate social environment can also change the innate preferences (Nekitsing et al. 2018).

All in all, although the sample size of the investigated population should be increased in future studies, taste perception seems to have an influence on caries activity in children. This requires continued efforts in terms of education and intensification of preventive measures. In this context, it would be interesting to find out whether changing the diet of children with caries as a result of parental education about dental health could bring about a change in the children's taste perception. Studies in adults show that lower sugar intake affects the intensity of sweet taste (Berthoud and Zheng 2012; Wise et al. 2016). There is evidence that decayed teeth, poor oral hygiene, and high exposure to oral bacteria such as acidogenic and acidophilic lactobacilli and streptococci affect the overall assessment of taste (Solemdal et al. 2012). It would also be of interest to investigate in a follow-up study whether the taste perception of children is influenced by changes in the oral microbiome after complete dental restoration under general anaesthesia. In this regard, the influence of bioadhesion and biofilm formation on taste perception (Hannig et al. 2016) could be further explored.

## Conclusions

Considering the limitations of the present study, it has been shown that:

– Taste perception in children appear overall associated with the presence of untreated carious teeth.
– The perception of the taste quality “bitter” was significantly worse in the children with caries compared to caries-free children.
– More studies are required to investigate whether changing the diet of children with caries or restore all carious teeth would affect their taste perception.
